# When probiotics guidelines differ: a practical guide for clinicians and researchers

**DOI:** 10.1080/19490976.2026.2657049

**Published:** 2026-04-15

**Authors:** Geoffrey A. Preidis, Hania Szajewska, Marla Cunningham, Howard Bauchner, Daniel J. Merenstein

**Affiliations:** a Department of Pediatrics, Division of Gastroenterology, Hepatology & Nutrition, Baylor College of Medicine and Texas Children’s Hospital, Houston, Texas, United States; b USDA/ARS Children’s Nutrition Research Center, Baylor College of Medicine, Houston, Texas, United States; c Department of Paediatrics, The Medical University of Warsaw, Warsaw, Poland; d International Scientific Association for Probiotics and Prebiotics, Brisbane, Queensland, Australia; e Boston University Chobanian & Avedisian School of Medicine, Boston, Massachusetts, United States; f Department of Family Medicine, Georgetown University Medical Center, Washington, DC, United States

**Keywords:** Certainty of evidence, necrotizing enterocolitis, network meta-analysis, systematic reviews

## Abstract

Many professional organizations publish clinical practice guidelines for the use of probiotics in gastrointestinal disorders. Generally, no two guidelines align perfectly, and some differ markedly. These discrepancies occur because clinical practice guidelines are not purely mechanical outputs of data synthesis. They are shaped by structured interpretation, value judgments, and predefined thresholds for uncertainty. These elements become particularly visible when effect sizes are small, heterogeneity is high, and outcomes vary in clinical relevance–as is the case with probiotic research. This article examines why discordance among probiotic guidelines is predictable and provides practical guidance for clinicians who must decide among conflicting recommendations to make the most appropriate management decisions for their patients. Guidance is also provided for investigators who seek to improve the quality of evidence, confidence in recommendations, and accurate communication of probiotic research to clinicians, patients, and the broader scientific ecosystem.

## The roles of clinical practice guidelines

In the practice of modern medicine, clinical decision-making requires rapid synthesis and interpretation of a continuously expanding body of evidence. To navigate this landscape, many clinicians rely upon guidelines published by trusted professional societies. Clinical practice guidelines assimilate available evidence and provide recommendations addressing the benefits and harms of different therapies for specific patient populations. Guidelines that are high quality can improve the safety, consistency, and quality of medical care. Yet, guidelines must be used cautiously and with appropriate context.

Rarely is the evidence that informs a clinical practice guideline incontrovertible. Observational studies are of variable quality, interventions often produce inconsistent results from one trial to another, and the validity and generalizability of many studies is limited by bias, imprecision, and inconsistency. These and other factors are assessed by carefully developed evaluation tools including Grading of Recommendations, Assessment, Development and Evaluation (GRADE).^1^ Well done guidelines identify data through an appropriate and complete database search, incorporate rigorous evaluation tools, and communicate the level of confidence the panel has in a recommendation. Additionally, well done guidelines acknowledge the limitations of the evidence, tempering the “hype” generated by news outlets and social media regarding the significance and potential impact of primary research findings.

Clinical practice guidelines are not simply numerical products of statistical algorithms. They are constructed using value judgments, predefined thresholds for uncertainty, and structured interpretation. These human elements become magnified in fields like probiotic research where the evidence and interventions are heterogeneous and effect sizes are modest. This article examines why discordance is common in probiotic guidelines and how improvements in study design, reporting, and scientific communication could increase the clinical impact and consistency of future guidelines.

## Reasons clinical practice guidelines differ

Even when high levels of rigor and transparency are maintained throughout the guideline development process, it is not unusual for different panels to examine a body of evidence and arrive at different conclusions. This scenario is common with probiotic guidelines. Over the past decade, numerous professional societies have published guidelines that address probiotics for gastrointestinal disorders, including the American College of Gastroenterology,^2^ American Gastroenterological Association (AGA),^3^ British Society of Gastroenterology (BSG),^4^ European Society for Pediatric Gastroenterology, Hepatology and Nutrition (ESPGHAN),^5^ World Gastroenterology Organization,^6^ and World Health Organization.^7^ Recommendations also exist for specific countries^8^^,^^9^ and regions of the world.^10^ Across many indications, these guidelines reach different conclusions or apply different thresholds for recommending probiotics, reflecting variation in scope, strain specificity, and certainty of evidence.

Guideline discordance for probiotics is not random. It reflects predictable differences in how probiotics are defined, evidence is synthesized, trials are selected, outcomes are prioritized, and uncertainty is judged. These factors are discussed below.

### Strain specificity

Several factors unique to probiotic research account for some of the discordance among guidelines. One unique factor is the heterogeneity of treatments that often are analyzed as a single intervention, “probiotics.” Probiotic products vary by genus, species and/or strain, and include different doses of single-strain or combination probiotics. Given the distinct biological properties of beneficial microbes (especially across genera and even kingdoms), combining all probiotics into a single treatment group risks obscuring biologically meaningful differences, increasing the risk of both false-negative and false-positive findings, and contributing to inconsistent guideline recommendations. We recommend avoiding pooling distinct probiotic strains into a single treatment arm unless there is a clear rationale (e.g., shared mechanisms of action, similar functional properties, or comparable clinical effects with substantial heterogeneity). In some cases, there are shared strain-specific—or even species-level—effects that justify combining different products within a meta-analysis. Examples of shared mechanisms include short-chain fatty acid production and pathogen exclusion.^11^

A second factor related to strain specificity is how meta-analyses combine data from different probiotic strains. This decision is important because only a small number of studies have been performed with each individual product. For example, AGA’s technical review in 2020 found that for adults and children with irritable bowel syndrome, 76 published randomized controlled trials (RCTs) tested a total of 44 different probiotic strains or combinations; for the majority of studies that reported a benefit, the totality of evidence was a single RCT.^12^ A conventional pair-wise meta-analysis compares each probiotic intervention to placebo, pooling head-to-head data for each individual treatment. Alternatively, a network meta-analysis leverages that every RCT contains a comparison group, enabling construction of a network of treatments that can be used to make indirect comparisons between different probiotic therapies that have not been tested head-to-head. By combining direct and indirect evidence, a network meta-analysis allows one to estimate how different treatments might compare with one another. Published probiotic guidelines have used various combinations of these approaches.

### Subjective elements of the GRADE approach

The GRADE approach provides a structured framework for assessing the certainty of evidence and translating that evidence into recommendations^1^^,^^13^ (Table 1). GRADE rates certainty across four levels (Table 2) based on clearly defined domains—risk of bias, inconsistency, indirectness, imprecision, and publication bias—and then considers the balance of benefits and harms, patient values, resource use, and feasibility when formulating a recommendation. Although GRADE supports transparency and consistency, it does not eliminate subjectivity. Panels may differ in how they downgrade for imprecision or heterogeneity, how they interpret the clinical importance of small effects, or how they weigh safety concerns when data are limited. Likewise, deciding whether the evidence supports a strong or a conditional recommendation is still a matter of judgment. As a result, different groups applying the same GRADE framework to the same evidence may reach different conclusions, contributing further to the variability observed across guidelines. It is also important to acknowledge that the body of evidence reviewed is unlikely to be identical between panels due to differences in database search timeframes and inclusion/exclusion criteria applied.^14^

**Table 1.** Interpretation of strong and conditional recommendations in the GRADE framework.

Recommendation	Interpretation	Implications for practice
Strong for	High confidence that benefits outweigh harms	Most patients in this situation should be offered the intervention and would likely choose it.
Weak (conditional) for	Benefits *probably* outweigh harms	Different choices may be appropriate for different patients. Clinicians should support patients in making decisions aligned with their values and preferences.
Weak (conditional) against	Harms *probably* outweigh benefits	Clinicians should discuss the uncertainty and explore patient preferences; some patients may still reasonably choose the intervention, but many will not.
Strong against	High confidence that harms outweigh benefits	Most patients in this situation should not be offered the intervention and would likely choose to avoid the intervention

**Table 2.** Rating the quality of evidence in the GRADE framework.

Quality level	Definition
High	We are very confident that the true effect lies close to that of the estimate of the effect.
Moderate	We are moderately confident in the effect estimate: The true effect is likely to be close to the estimate of the effect, but there is a possibility that it is substantially different.
Low	Our confidence in the effect estimate is limited: The true effect may be substantially different from the estimate of the effect.
Very Low	We have very little confidence in the effect estimate: The true effect is likely to be substantially different from the estimate of effect.

Disagreement between guideline panels applying GRADE is expected when effect sizes are small, heterogeneity is high (data derived from observational studies vs RCTs), or outcomes differ in clinical relevance. Persistent discordance across multiple indications more often reflects fragile or incomplete evidence rather than failure of the GRADE framework.

### Why guidelines differ—a case study

One pair of differing probiotic recommendations is particularly illustrative. Despite relying on similar methodologies, including paired strain-specific systematic reviews, network meta-analyses, and the GRADE framework to assess the quality of evidence, AGA and ESPGHAN published contrasting recommendations regarding the role of probiotics in the management of gastrointestinal disorders.^3^^,^^5^ Of the eight gastrointestinal conditions for which one or both groups determined that enough evidence existed to issue a recommendation, the two guidelines never agree completely (Table 3). Among the notable differences, for children with acute gastroenteritis, AGA recommends against using probiotics whereas ESPGHAN recommends one of four probiotic treatments, and for the prevention of serious gastrointestinal disorders in preterm and low birth-weight infants, AGA recommends one of 13 probiotic strains or combinations whereas ESPGHAN recommends using one of two treatments and recommends against using two other strains of probiotics (Table 4).

**Table 3.** Comparison of AGA 2020 and ESPGHAN 2023 probiotic recommendations.

Clinical Indication	AGA 2020	ESPGHAN 2023
Acute gastroenteritis	Conditional recommendation against probiotic use (moderate certainty).	Conditional recommendation for: *Lacticaseibacillus rhamnosus* GG ATCC 53103 (low certainty); *Saccharomyces boulardii* (low certainty)*; Limosilactobacillus reuteri* DSM 17938 (very low certainty); or *L. rhamnosus* 19070-2 + *L. reuteri* DSM 12246 (very low certainty). Strong recommendation against: *Lactobacillus helveticus* R0052 + *L. rhamnosus* R0011 (moderate certainty). Conditional recommendation against: *Bacillus clausii* strains (very low certainty).
Prevention of antibiotic-associated diarrhea	–	Strong recommendation for: *S. boulardii;* or *L. rhamnosus* GG ATCC 53103 (moderate certainty).
Prevention of nosocomial diarrhea	–	Conditional recommendation for: *L. rhamnosus* GG ATCC 53103 (moderate certainty). Strong recommendation against: *L. reuteri* DSM 17938 (high certainty).
Prevention of *C. difficile* infection during antibiotic therapy	Conditional recommendation for: *S. boulardii*; *Lactobacillus acidophilus* CL1285 + *Lacticaseibacillus casei* LBC80R; *L. acidophilus* + *Lactobacillus delbrueckii* subsp. *bulgaricus* + *Bifidobacterium bifidum*; or *L. acidophilus* + *L. delbrueckii* subsp. *bulgaricus* + *B. bifidum* + *Streptococcus thermophilus* (low certainty).	–
*C. difficile* infection—treatment	Only in the context of a clinical trial (knowledge gap)	–
Crohn’s disease	Only in the context of a clinical trial (knowledge gap)	Insufficient evidence
Ulcerative colitis	Only in the context of a clinical trial (knowledge gap)	Insufficient evidence
Pouchitis	Conditional recommendation for: *Lacticaseibacillus paracasei* subsp. *paracasei* + *Lactiplantibacillus plantarum* + *L. acidophilus* + *L. delbrueckii* subsp. *bulgaricus* + *Bifidobacterium longum* subsp. *longum* + *Bifidobacterium breve* + *B. longum* subsp. *infantis* + *S. thermophilus* (very low certainty).	–
Irritable bowel syndrome (IBS)	Only in the context of a clinical trial (knowledge gap)	–
Functional abdominal pain/IBS	–	Conditional recommendation for: *L. reuteri* DSM 17938; or *L. rhamnosus* GG ATCC 53103 (moderate certainty).
Functional constipation	–	Conditional recommendation against probiotic use (moderate certainty).
Infant colic	–	Conditional recommendation for: *L. reuteri* DSM 17938; or *Bifidobacterium animalis* subsp. *lactis* BB-12 (moderate certainty).
*Helicobacter pylori* infection (as adjuvant)	–	Conditional recommendation for: *S. boulardii* (very low certainty).
Preterm, low-birth-weight infants (see also Table 4)	Conditional recommendation for: *Lactobacillus* spp. + *Bifidobacterium* spp. (7 combinations); *B. animalis* subsp. *lactis*; or *L. reuteri* (2 strains); or *L. rhamnosus* (3 strains) (moderate/high certainty)	Conditional recommendation for: *L. rhamnosus* GG ATCC 53103; or *B. longum* subsp. *infantis* BB-02 + *B. animalis* subsp. *lactis* BB-12 + *S. thermophilus* TH-4 (low certainty). Conditional recommendation against: *B. breve* BBG-001; or *S. boulardii* (very low to moderate certainty)
Celiac disease	–	Insufficient evidence
Small intestinal bacterial overgrowth	–	Insufficient evidence
Pancreatitis	–	No data

**Table 4.** Comparison of probiotic recommendations for preterm, low-birth-weight infants: ESPGHAN 2020/2023 vs AGA 2020.

Reference	Outcomes	Probiotics recommended	Probiotics not recommended	No recommendation/insufficient evidence
ESPGHAN 2020 & 2023	Necrotizing enterocolitis	*L. rhamnosus* GG ATCC 53103 *B. longum* subsp. *infantis* BB-02 + *B. animalis* subsp. *lactis* BB-12 + *S. thermophilus* TH-4	*B. breve* BBG-001 *S. boulardii*	*L. reuteri* DSM 17938 *B. bifidum* NCDO 1453 + *L. acidophilus* NCDO 1748
AGA 2020	All-cause mortality Necrotizing enterocolitis Culture proven sepsis Feed intolerance Days to reach full feed Days of hospitalization	Combinations of *Lactobacillus* spp. and *Bifidobacterium* spp., including: ∘ *L. rhamnosus* GG ATCC 53103 + *B. longum* subsp. *infantis* ∘ *L. casei* + *B. breve* ∘ *L. rhamnosus* + *L. acidophilus* + *L. casei + B. longum* subsp. *infantis* + *B. bifidum* + *B. longum* subsp. *longum* ∘ *L. acidophilus* + *B. longum* subsp. *infantis* ∘ *L. acidophilus* + *B. bifidum* ∘ *L. rhamnosus* GG ATCC 53103 + *B. longum* Reuter ATCC BAA-999 ∘ *L. acidophilus* + *B. bifidum* + *B. animalis* subsp. *lactis* + *B. longum* subsp. *longum* **B. animalis* subsp. *lactis*, including:* ∘ *DSM 15954* *L. reuteri*: ∘ DSM 17938 ∘ ATCC 55730 *L. rhamnosus*: ∘ ATCC 53103 ∘ ATC A07FA	None specifically listed as “not recommended” or “insufficient evidence,” but numerous strains and combinations judged to have low or very low certainty of evidence	

Several methodological differences described in the guideline development processes likely contribute to the discrepant recommendations of AGA and ESPGHAN. Guideline development panels apply different thresholds of evidence for incorporation into recommendations. In this case, ESPGHAN inclusion criteria required at least two RCTs testing a probiotic treatment for a given condition, whereas AGA excluded all open-label studies. Geography also played a role, with AGA placing increased weight on evidence generated in North America, where most of its members practice medicine.^15^ This emphasis is particularly important for acute gastroenteritis, given that infectious etiologies vary by global region. Finally, relevant to preterm and low birth-weight infants, AGA evaluated evidence for six outcomes (all-cause mortality, necrotizing enterocolitis, culture proven sepsis, feed intolerance, days to reach full feed, and days of hospitalization) and considered strains that could prevent any of these comorbidities of prematurity, whereas ESPGHAN focused on prevention of necrotizing enterocolitis. Although both organizations issued conditional recommendations supporting the use of specific probiotics, ESPGHAN generally assigned lower certainty ratings, whereas AGA acknowledged the limitations but rated the evidence for selected probiotic combinations as moderate to high certainty. This was based on the large magnitude of effect in the network meta-analysis (e.g., 65% reduced odds of necrotizing enterocolitis with select combinations of *Lactobacillus* spp. + *Bifidobacterium* spp.) and opposing plausible residual bias (one RCT that assessed probiotic colonization found that nearly half of infants in the placebo arm were inadvertently colonized,^16^ potentially confounding results by reducing the true magnitude of effect).

Other reasons for these discrepancies are more complex, as discussed by Weizman and Vandenplas.^17^ Objective factors include variation in how panels select and appraise trials, apply thresholds for risk of bias and heterogeneity, and judge the relevance of older or smaller studies. Subjective factors include conflicts of interest bias, different opinions of expert panel members, and differing personal, intellectual, or emotional perspectives.^17^ Panelists might disagree on how much uncertainty is acceptable, on what constitutes a clinically meaningful effect, and on how much emphasis to place on feasibility and safety. Together, these methodological and judgment-based elements account for why panels reviewing comparable evidence reach conflicting conclusions. This case study demonstrates how modest differences in methodology and judgment can result in different recommendations in probiotic research, even when panels review similar evidence. Similar discordance is also observed in adult gastroenterology. For example, while AGA guidelines do not recommend probiotics for irritable bowel syndrome (IBS), BSG guidelines suggest that “probiotics, as a group, may be an effective treatment for global symptoms and abdominal pain in IBS.”^4^

## Interpreting guideline recommendations

A strong recommendation indicates high confidence that benefits outweigh harms (or vice versa). In clinical practice, this means that most patients would choose the intervention, clinicians are expected to offer it routinely, and policymakers may rely on such recommendations when developing standards of care. In contrast, a conditional recommendation indicates greater uncertainty and acknowledges that different choices may be appropriate for different individuals.

Conditional recommendations, whether for or against, place greater emphasis on shared decision-making. Clinicians should explain the magnitude and relevance of any potential benefit; discuss uncertainties and safety considerations; and explore patient or family preferences and values, the cost of the intervention, and expectations before deciding whether to use a probiotic or any other intervention. This approach is especially important when the evidence is less clear or the expected benefit versus risk is small. Notably, the practical interpretation of a conditional recommendation “for” and a conditional recommendation “against” is largely similar. Thus, in the case of children with acute gastroenteritis, both AGA’s conditional recommendation against probiotic use and ESPGHAN’s conditional recommendation to use specific probiotic strains require clinicians to help patients and their families make a decision that is consistent with their own values.

## Increasing the impact of future probiotic research

Clinicians seek clinical data generated in settings that reflect real-world practice, ensuring strong external validity and clear relevance to the patients they treat every day. They prioritize studies that measure outcomes that genuinely matter to patients and clinicians,^18^ often referred to as PROM (Patient-Reported Outcome Measures). In a field like probiotic research where products developed as dietary supplements rarely have large research and development budgets, not every intervention will have drug-level evidence, although that is strongly preferred. When only a limited number of outcome measures is possible, it is important that clinical outcomes are prioritized and measured accurately. Patient-centered outcomes should take precedence over disease- or biomarker-focused outcomes. For example, a patient’s clinical improvement in ulcerative colitis is more important than a positive shift in their Shannon index of fecal microbial community diversity. Similarly, weaning a patient from respiratory support during influenza infection is more important than the patient’s viral load.

External validity is important in all aspects of clinical research,^19^^,^^20^ but might be even more integral to interventions like probiotics that can be influenced by the host microbiome. When an intervention’s effect is theorized to be dependent on the microbiome, it is essential to evaluate whether the study population accurately reflects the population in which the product is intended. Thus, a probiotic with proven efficacy to prevent diarrhea in rural India may have little effectiveness in a hospitalized patient in Italy. While it is important to collect PROM data, these data should complement other clinically relevant and validated outcomes, and, where possible, standardized PROM measures should be used to improve comparability across studies and facilitate guideline development. Similarly to antibiotics, specificity is important for probiotics, and clearly identifying strain and dose is essential to help establish a strong evidence base.^21-23^ Conducting follow-up studies to replicate findings for a specific strain and dose is also critical given that a key barrier for evidence-based guideline synthesis is the variety of different strains being studied and the small number of studies testing each. Meanwhile, sometimes identical strains are studied but at variable doses and durations, potentially even at higher doses than are commercially available.

Collecting and reporting adverse event data is especially important because safety concerns are often underreported. This is a particular problem for probiotic research, as it is often falsely assumed that probiotics are inherently safe. A recent individual patient data meta-analysis (IPDMA) of 35 studies found that 16 studies provided no adverse event data and of the remaining 19, eight stated that zero adverse events occurred.^24^ This type of data collection and reporting highlights a major gap that undermines trust and limits clinical applicability. Ultimately, clinicians want evidence that is rigorous, transparent, and directly usable to guide safe and effective patient care.

It is possible for large studies to find statistically significant differences−with impressively low *p*-values−yet result in minimal clinical benefit.^25^ In the probiotics field, the opposite problem is common: studies are typically small, fail to reach statistical significance, and are prematurely dismissed as ineffective, yet if the sample size had been larger, a clinically significant effect might have been observed. This dynamic likely leads to an excess of Type II errors, masking potentially meaningful effects. It is also a key reason why many investigators are surprised when meta-analyses, which aggregate these underpowered studies, reveal more robust outcomes than expected. Sufficiently powering studies can be expensive, but funders and researchers need to be reasonable with expectations and effect sizes.

Finally, every member of the scientific ecosystem can contribute to a more rigorous and impactful future of probiotic research through responsible communication. Ecosystem members include federal agencies, professional societies, foundations, insurers, industry and venture capital, mainstream media, social media, and journals. The scientific communication ecosystem is vast, growing, and contributing to public mistrust in science, physicians, and researchers. Although only a handful of scientific articles published each year truly change clinical practice with little debate, the use of words that represent hype in published abstracts funded by the National Institutes of Health increased roughly 1404% between 1985 and 2020.^26^ The reasons for this are clear: investigators want their work acknowledged and appreciated (stable work and academic promotion often depend on this), medical journal editors aim to increase citations, reputation and financial viability, and news outlets seek views and website clicks. Both qualified and unqualified experts who debate in the public domain have no shortage of controversial topics, including the health risks of moderate drinking, use of body mass index to assess weight, the medical treatment of obesity in children, and the roles of probiotics in gastrointestinal disorders. As discussed above, both AGA’s and ESPGHAN’s conditional recommendations were similar, but how this message is relayed can appear very differently.

Responsible scientific communication^27^ requires investigators to pre-register study protocols, avoid statements that reflect causality when mere associations are observed, and avoid extreme language including “first of its kind” or “transformative.” Further, researchers should emphasize primary results rather than subgroup analyses, honestly convey effect sizes using easily understandable metrics including number needed to treat, present both absolute and relative differences, and review post-publication press releases to ensure that media outlets are not adding undue hype. Similarly careful language should be used when presenting results at scientific meetings or related venues. Experts are encouraged to join debates over challenging and controversial topics to provide important perspectives which can guide clinicians, patients, and other stakeholders. Opinions can be provided as published viewpoints, perspectives, editorials, podcasts, or interviews with media outlets, yet experts must be aware of their own biases, acknowledge alternative perspectives, and disclose all conflicts of interest. Responsible communication along all these nodes of the scientific ecosystem is a step in the direction toward stronger and more clinically impactful clinical practice guidelines.

## Conclusion

Disagreement among probiotic guidelines is not a failure of evidence-based medicine but a predictable consequence of fragmented data, strain heterogeneity, and subjective judgment. Guidelines should be used as decision-support tools, not as definitive answers. More consistent guidance can be achieved through higher quality, more transparent, and clinically relevant probiotic research that is communicated clearly, effectively, and honestly.
